# Supplementary feeding of birds during the winter influences measures of avian community structure in yards in a subtropical city

**DOI:** 10.1371/journal.pone.0302007

**Published:** 2024-05-22

**Authors:** Amanda M. Lamberson, Jennifer A. Smith

**Affiliations:** Department of Integrative Biology, The University of Texas at San Antonio, San Antonio, Texas, United States of America; UFERSA: Universidade Federal Rural do Semi-Arido, BRAZIL

## Abstract

Supplementary feeding, the intentional provision of food to wild birds is a common activity in developed nations during the winter. The energy inputs represented by supplementary feeding are vast, and thus it is likely an important mechanism shaping bird communities in urban areas. However, research in this regard has mainly occurred in temperate and non-urban settings. Moreover, few studies have been informed by supplementary feeding habits of local community members limiting their inference. We evaluated the effects of two commonly provided wild bird foods on the abundance and species diversity of birds in yards over two winters in San Antonio, Texas, United States, a city located in a subtropical region. We used a reversed Before-After-Control-Impact experimental design in which yards were randomly allocated either mixed seed, Nyjer, or no food (control) between November 2019 and March 2020 (Year One). Between November 2020 and March 2021 (Year Two) supplementary food was not provided in any yards. Point counts conducted during both years of the study revealed that overall bird abundance was consistent between years in control yards and yards provided with Nyjer. In contrast, overall bird abundance was statistically significantly higher when supplementary food was present in mixed seed yards, driven by an increase in granivorous and omnivorous species. In contrast, supplementary feeding had no statistically significant effect on the abundance of insectivorous species or on species diversity, although species diversity tended to be higher in the presence of mixed seed. Our study demonstrates that wild bird food commonly provided by community members influences measures of avian community structure during the winter in urban yards in a subtropical city. However, these results depend on the type of bird food provided. Our results provide insight into the processes underlying the effects of urbanization on bird communities, and thus have implications for the management of urban birds more broadly.

## Introduction

Supplementary feeding, the provision of food by humans that provides a subsidy to the natural diets of wild birds, is a widespread phenomenon in urban areas in much of the developed world [1–3]. While supplementary feeding may be unintentional (e.g., via food waste), we focus on the intentional provision of food (e.g., via backyard bird feeders). In the United States (US) in 2016, estimates suggested 70% of 81.1 million participants of around-the-home activities intentionally fed wild birds at some point during the year, spending approximately US $4 billion annually on bird food [4]. Similarly, in 2008 it was estimated that US $220 million and US $440 million were spent annually on bird food in mainland Europe and in the United Kingdom, respectively [2]. The energy inputs into backyards represented by such extensive feeding and the number of birds likely supported are staggering [5]. Not surprisingly, the potential effects of supplementary feeding on birds are a continuing topic of scientific inquiry [3,6,7].

Previous research suggests that supplementary feeding has myriad effects on wild birds including on breeding phenology and measures of breeding productivity [8–11], breeding behaviors [12,13], and disease transmission [14–17]. In accordance with optimal foraging theory which posits that animals should select foraging sites that provide the greatest energetic and nutritional net gain (e.g., those in which prey is relatively predictable, where prey encounter rate is high, and where prey handling time is low [18,19]), supplementary feeding should also increase the presence of birds, and thus abundance (the total number of individual birds present) and species diversity (a measure that considers the number of species and species evenness) of birds because it provides a predictable and reliable food source. This has been demonstrated by numerous correlational studies [20,21]. However, to our knowledge only one study has experimentally assessed the effects of supplementary feeding on the abundance and species diversity of birds [22] limiting our capacity to understand the importance of supplementary feeding as a mechanistic driver of avian community structure in urban areas. Given that key motivations for providing wild bird food are to attract birds and to experience nature [23,24], additional studies in this regard are also needed to inform members of the public who feed birds.

While supplementary feeding may increase avian abundance [20,22], responses to supplementary feeding are likely taxa-specific, in part underscored by diet. Seeds and grains are some of the most common food types intentionally provided to wild birds [1,5]. Thus, increased avian abundance in response to supplementary feeding may be explained by higher incidences of granivores and omnivores [22]. In contrast, insectivores may decrease after being displaced by species attracted to bird feeders [22]. The type of food provided may also affect species presence at feeders, e.g., Nyjer typically attracts smaller finches (*Carduelis* spp.) whereas black-oil sunflower attracts chickadees (*Poecile* spp.) and larger finches (*Carpodacus* spp.) [25]. Despite these ideas, experimental approaches investigating taxa-specific responses to different supplementary food types in this regard are absent.

Of the studies evaluating the effects of supplementary feeding on birds, many have focused on birds in non-urban areas [11,13,26], with only a few focusing on urban settings, mostly in temperate regions [5,22,27]. Results of these studies may not be relevant in subtropical regions due to geographic variation in environmental drivers of foraging behavior (e.g., climate, resource availability) [28,29] and the composition and ecology of bird communities that may affect the outcomes of such studies. Thus, additional assessments of the effects of supplementary feeding on birds in urban areas in subtropical regions are needed. Such studies are especially relevant during the winter, when supplementary feeding is often more common than in other seasons [1,5,30].

This project aimed to evaluate the effects of two commonly provided wild bird foods, mixed seed and Nyjer on the abundance and species diversity of birds in yards in San Antonio, Texas, US during the winter. San Antonio is the 7th most populated US city and one of the fastest growing [31]. It has a subtropical climate characterized by hot summers and mild winters. Thus, San Antonio affords a unique opportunity to address critical missing gaps in knowledge centered on the effects of supplementary feeding on birds. We used a reversed Before-After-Control-Impact (BACI) experimental design to evaluate our hypothesis that the abundance and species diversity of birds would be higher in yards provided with supplementary food compared to in yards without supplementary food because of the increased availability and predictability of resources. Further, we predicted that yards with mixed seed would have a higher abundance and greater species diversity compared to those with Nyjer because Nyjer would be limited to species adapted to eating smaller seeds. We also hypothesized that the provision of mixed seed and Nyjer would have a greater effect on the abundance of granivores and omnivores, compared to insectivores due to their dietary focus on seeds and grains.

## Materials and methods

### Study area

Our study was conducted in the northern half of San Antonio, (29° 25’N, 98°29’W), a large (1,194 km^2^) city located in Bexar County, Texas, US. Limiting the study to this area was done for the purpose of reducing potential effects of underlying ecological and socioeconomic variation across the city on the abundance and species diversity of birds (e.g., due to the luxury effect [32,33]). The study occurred in the transition area of the Texas Hill Country and Blackland Prairie ecoregions, an area characterized by many scattered parks and green spaces embedded in an urban matrix. Vegetation includes native trees and shrubs (e.g., live oak [*Quercus fusiformis*], Texas red oak [*Quercus texana*]) and non-native ornamentals [34]. Average monthly high and low temperatures during the focal period for our study (November-March) are 15.6°C to 21.1°C, and 4.4°C to 10°C, respectively [35].

### Experimental design

The effect of supplementary feeding on the abundance and species diversity of birds in yards was evaluated using a reversed BACI study design during two subsequent winter field seasons: November 11, 2019, to March 5, 2020 (hereafter ‘Year One’), and November 14, 2020, to March 7, 2021 (hereafter ‘Year Two’). We recruited and gained permission to access 36 urban, private residential backyards (hereafter ‘yards’) through written advertisement and public outreach events. Yards were at least 500 m apart (average size: 0.05 ha; range: 0.02–0.23 ha) and all but two were ≥1000 m apart, such that yards were independent based on the average winter home range of the northern cardinal (*Cardinalis cardinalis*) (21.20 ha) [36], and the maximum winter home range of the house sparrow (*Passer domesticus*) (2.90 ha) [37], two species common at bird feeders in San Antonio. This approach aligns with that of previous studies who considered between-yard distances between 300 m and 500 m to be sufficient to obtain site independence [20,22,33]. Each yard was required to have at least one tree present to provide cover for birds.

A previous assessment of bird food sales in San Antonio suggests that mixed seed and Nyjer seed are sold in larger quantities by weight than other types of wild bird food in the area [38]. As a result, mixed seed and Nyjer seed (Wild Birds Unlimited, IN, US) were selected for this study. The variety of mixed seed used (‘Supreme Blend’) contained black-oil sunflower, sunflower chips, safflower, and striped sunflower seeds. Nyjer was a pure mix with no other additions. The 36 yards were equally and randomly allocated to one of three treatment groups: (1) mixed seed, (2) Nyjer, or (3) control (non-fed yards). During Year One, food was available continuously in a single feeder at fed yards, whereas control yards were not provided with a feeder, nor did they receive supplementary food. Mixed seed was provided via a 1.10 kg capacity Brome® Squirrel Buster™ (Brome Bird Care, QC, Canada) and Nyjer was provided via a 0.70 kg capacity Perky-Pet® finch feeders (Woodstream Corporation, Inc., PA, US). In October 2019, prior to the onset of data collection in Year One, feeders were mounted on 2.10 m shepherd’s hooks or hung from a tree branch at least 1.50 m off the ground. During Year Two no yards received supplementary food. At the beginning of each field season, residents of focal households were asked to cease feeding at least two weeks prior to the start of data collection to minimize the effects of existing feeding on bird abundance and species diversity. Hummingbird feeders were allowed to stay up at the resident’s request as it was deemed unlikely that hummingbirds (*Trochilidae* spp.) or the nectar feeders would affect birds visiting experimental feeders.

### Bird surveys

Point counts were conducted in each yard to evaluate bird abundance and species diversity. Point count locations (one per yard) were selected that maximized (1) visibility of the focal yard (the area enclosed by the property line), and (2) the distance between the observer and the feeder (in fed yards). From each location, we conducted 15-minute point counts in which all birds seen or heard within the yard were identified to species and counted using binoculars. Bird species counts were based on the greatest number of individuals seen at one time during the count to prevent double counting of the same bird. Birds flying over without stopping and birds of prey were not counted. Before starting each point count, we included a five-minute settling period to allow disturbed birds to return and acclimatize to the observer’s presence. Observers recorded date, time, and ambient temperature at the start of each point count. Temperature was recorded using an outdoor thermometer, or a phone app reporting local weather conditions.

Point counts were conducted in each yard once every two weeks by lab personnel during both field seasons. Yards surveyed within a day (typically three or four) were grouped based on geographic proximity to facilitate fieldwork. Within a day, each yard within a group was allocated a point count start time of either 0730, 0815, 0900, or 0945 (Central Standard Time) to minimize any potential daytime-specific bias [39]. Within each two-week survey period the day of the week on which each group of yards had point counts conducted was rotated to minimize the potential for day-specific factors to bias data (e.g., disturbance associated with regular yard work, construction, garbage collection, or others [22]). In the event of inclement weather (winds exceeding 26 km/h or heavy rain), point counts were postponed until the next suitable day.

Additional point counts were conducted by residents of focal households who participated as citizen scientists [40,41], and who followed a modified version of the protocol described above. Specifically, they conducted point counts as previously described but from an indoor vantage point where they could view most of the yard. Citizen scientists conducted point counts weekly and were requested to select a consistent time to survey. Datasheets and binoculars were provided to all participants. Resources were provided to citizen scientists to familiarize them with the most common bird species. In January 2020 a multiple-choice quiz was given to assess their ability to identify birds by sight. On average, the 26 citizen scientists who participated in the quiz got 80% (SD = 0.15) of the 16 questions correct. Based on quiz results, an identification guide was developed and distributed to citizen scientists. In November 2020, a new quiz was administered to citizen scientists who elected to continue in Year Two, in addition to lab personnel (six undergraduates) who assisted with data collection in Year Two. On average, the 14 participants of the quiz got 76% (SD = 0.18) of the 16 questions correct. Results were shared with citizen scientists and lab personnel who were encouraged to review training materials as needed. Approval for this research was received by The University of Texas at San Antonio’s Institutional Animal Care and Use Committee (IACUC; protocol #BI001-08/22).

### Landscape variables

The presence of water sources (e.g., bird baths) was recorded in each yard to account for their potential effect on bird abundance and species diversity [42]. Similarly, we estimated the percentage of impervious cover within a 500-m radius of each yard using ArcGIS Pro 2.5.1 (ESRI, Redlands, CA, US) and an impervious cover shapefile [43] to account for the potential effects of urban land cover on our results [44]. We considered a 500-m radius as it encompasses the winter home range of northern cardinals [36], house sparrows [37], and presumably other common feeder species in our study area whose presence may be influenced by urban land cover.

We also estimated feeder density within a 100-m buffer of each focal yard via door-to-door surveys distributed to households in winter 2020/2021 (see [38]). We constrained surveys to within a 100-m buffer of each focal yard as this is the area we considered most likely to be used by birds observed during the point counts. In brief, we developed a survey that requested information about the number of bird feeders (excluding hummingbird feeders) used by each household during our study period. Surveys were delivered to all households within each 100-m buffer using a door-to-door approach that considered social distancing (i.e., due to the COVID19 pandemic). Feeder density was calculated as the number of reported active feeders per 100 m radius of households surrounding the focal yard. Prior to data collection, survey methods were approved by The University of Texas at San Antonio’s Institutional Review Board (IRB; protocol #19-247E). All participants reviewed a written consent form prior to taking part in the survey.

### Statistical analysis

Mixed-effects models were constructed to evaluate the effects of supplementary feeding in yards on (1) the overall abundance of birds, (2) the abundance of birds by dietary guild (granivorous, omnivorous, insectivorous), and (3) species diversity using package lme4 [45] in R (2022, version 4.2.2). Abundance was considered as the total number of birds seen or heard per point count. Species diversity was determined for each point count using Shannon’s Diversity Index (H’), a measure that considers both species richness (the number of different species detected within a defined area) and species evenness (the distribution of abundance across all species within a defined community). This index was selected as it is equally sensitive to both rare and abundant species [46]. Birds were assigned to dietary guilds based on their winter diet and foraging behavior (birdsoftheworld.org [see supporting information]).

The effects of supplementary feeding in yards on (1) the overall abundance of birds, and (2) the abundance of birds by dietary guild were assessed separately using generalized linear mixed models (GLMMs) fitted with Poisson error distributions. For each analysis, we developed a set of candidate models, all of which contained treatment (mixed seed, Nyjer, control), year (Year One or Year Two), and the interaction between treatment and year as fixed main effects. Yard site ID was included as a random effect to account for repeated measures at each yard. In addition, we considered a suite of covariates to control for their potential impact on bird abundance: the density of feeders within a 100-m buffer of the focal yard [20], water feature presence in the focal yard [42], temperature [44], percentage of impervious cover within a 500-m buffer of the focal yard [20,44], and start time of the point count [39]. We did not consider observer type (i.e., lab personnel, citizen scientist) as a covariate because preliminary analysis of the bird quiz data suggested that bird identification ability was consistent amongst observers. Prior to developing models, we used Spearman’s Rank correlation to assess multicollinearity between potential explanatory variables and removed one of two correlated variables where *r*_*s*_ ≥ 0.5 to minimize the potential effects of multicollinearity [47]. We assessed the global models for each candidate set for overdispersion using Pearson’s Chi-squared test in the AER package [48]. Due to overdispersion, we subsequently fit all abundance models with a negative binomial error distribution.

We evaluated the effects of supplementary feeding on species diversity using linear mixed models (LMMs) fitted with a normal error distribution. We developed a candidate set of models that considered the same main fixed effects, random effect, and suite of uncorrelated covariates used for the abundance models. Visual inspection of a QQ plot and results of Shapiro-Wilk test (P = 0.08) confirmed model residuals were normally distributed.

We ranked models in each candidate set and selected the model that received the most support from the data using Akaike’s Information Criterion corrected for small sample size (AIC_c_). We estimated ΔAIC (AIC_c_ model–AIC_c_ min) and AIC_c_ weights (w_*i*_) to facilitate model selection using the R package MuMIn [49]. The top model which received the most support from the data was considered as the one with the lowest ΔAIC_c_ score. Competing models were considered those with ΔAIC_c_ scores of ≤ 2.00 [50] unless they contained an uninformative parameter [51]. We considered a parameter uninformative if its addition to a simpler model (i.e., a model with less parameters) resulted in ΔAIC_c_ ≤ 2, and thus where it did not explain enough variation in the data to justify its inclusion. For brevity, we only report informative models. Model estimates are presented as untransformed estimates unless otherwise stated. We considered model estimates statistically significant where the 85% confidence interval did not include zero [52]. The use of 85% confidence intervals was considered to allow for a more congruent evaluation of model parameter estimates, a recommended approach when competing models are similar in structure and have similar AIC_c_ values [51,53].

## Results

In Year One (i.e., November 2019 –early March 2020), point counts were conducted in 36 yards with 12 allocated to each treatment group. However, in Year Two (i.e., November 2020 –early March 2021) 13 households dropped out due to the COVID19 pandemic. As a result, we were limited to conducting point counts in 23 of the 36 yards. Of the 13 yards that did not participate in Year Two, six were designated as mixed seed-fed yards, five as Nyjer-fed yards, and two as control yards. Due to these extenuating circumstances, the number of citizen scientists conducting point counts also decreased; of the 36 yards used in Year One, 27 were monitored by citizen scientists, and 10 of the 23 yards used in Year Two were monitored by citizen scientists. In Year One, 267 point counts were conducted by lab personnel and 346 were conducted by citizen scientists. In Year Two, 169 point counts were conducted by lab personnel and 124 were conducted by citizen scientists. Amongst field seasons, 6,088 birds from 40 species were counted (Year One: 4,470 from 36 species; Year Two: 1,618 from 32 species); five accounted for 55% of detections (lesser goldfinch [*Spinus psaltria*], house sparrow, white-winged dove [*Zenaida asiatica*], northern cardinal, and black-crested titmouse [*Baeolophus atricristatus*]). An additional 21 species each made up < 1% of the total birds counted (see S1 Table). The number of feeders within 100-m of each yards was 1.48 (range: 0.00 to 6.37 no./ha), while the average percentage of impervious cover within a 500-m radius around each yard was 40% (range: 16% to 69% of 78.50 ha). Point count start time was positively correlated with ambient temperature (r_*s*_ = 0.46, P < 0.001). Therefore, point count start time was not considered further during the modelling process to minimize the potential effects of multicollinearity on parameter estimates [47].

### Bird abundance

**Fig 1:**
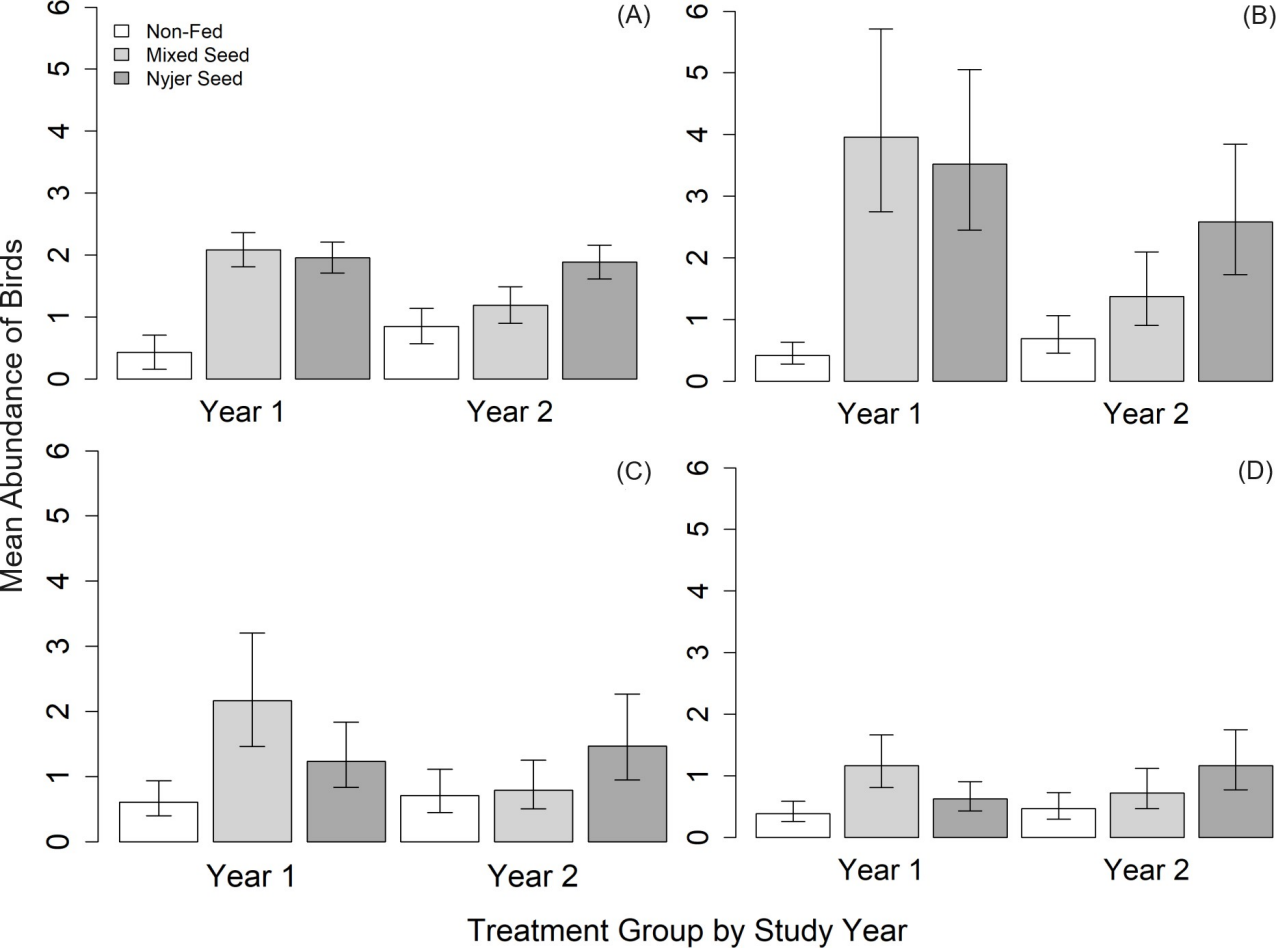
The abundance of birds in yards (mean ± 85% CI) in response to winter supplementary feeding. (A) Overall abundance and abundance by dietary guild ([B] granivorous, [C] omnivorous, [D] insectivorous), in Year One (November 2019 –early March 2020) when yards were either provided with mixed seed, Nyjer, or no food (control) and in Year Two (November 2020 –early March 2021) when all yards received no food. Estimates provided for the dietary guilds are back-transformed for visualization purposes.

Of the 13 models constructed to describe the effects of supplementary feeding on the overall abundance of birds in yards, the model containing the main effects of year and treatment, and the interaction between year and treatment received the most support from the data (Table 1). The estimates from the top-ranked model suggest that supplementary feeding had a statistically significant effect on the overall abundance of birds in yards. Specifically, the mean number of birds was significantly lower in mixed seed yards in Year Two when food was not provided compared to in Year One when food was provided (Year One: 2.08, 85% CI = 1.81–2.36; Year Two: 1.19, 85% CI = 0.90–1.49; Fig 1A). Overall, the mean number of birds remained similar in control yards between the two years of the study (Year One: 0.43, 85% CI = 0.16–0.71; Year Two: 0.85, 85% CI = 0.57–1.14; Fig 1A). Likewise, the mean number of birds was similar between years in Nyjer fed yards (Year One: 1.96, 85% CI = 1.71–2.21; Year Two: 1.89, 85% CI = 1.62–2.16; Fig 1A). In Year Two when no supplementary food was provided, while the mean number of birds was equivalent between control and mixed seed yards (control: 0.85, 85% CI = 0.57–1.14; mixed seed: 1.19, 85% CI = 0.90–1.49; Fig 1A), the mean number of birds was significantly higher in Nyjer fed yards compared to in both control and mixed seed yards (Nyjer: 1.89, 85% CI = 1.62–2.16).

**Table 1:** Models describing overall bird abundance, abundance by dietary guild, and species diversity in urban yards in San Antonio, Texas, US in response to winter supplementary feeding.

Variable	Model	*k*	AIC_c_	ΔAIC_c_	w_*i*_	LogLik
Overall Bird Abundance^a^	Base^b^	7	4909.80	0.00	0.23	-2446.80
	Null	2	4957.40	47.63	0.00	-2475.68
Abundance^a^ of Granivores	Base	7	3637.90	0.00	0.28	-1810.88
	Null	2	3693.60	55.70	0.00	-1843.80
Abundance^a^ of Omnivores	Base	7	3022.00	0.00	0.23	-1502.94
	Null	2	3044.20	22.12	0.00	-1519.07
Abundance^a^ of Insectivores	Base + Temperature + % Impervious Cover	9	2307.80	0.00	0.34	-1143.78
	Base + % Impervious Cover	8	2309.60	1.75	0.14	-1145.68
	Base + Temperature	8	2309.60	1.85	0.13	-1145.72
	Null	2	2326.80	19.00	0.00	-1160.39
Species Diversity^c^	Base + % Impervious Cover	8	1568.40	0.00	0.45	-775.08
	Base	7	1568.80	0.42	0.36	-776.31
	Null	2	1596.90	28.51	0.00	-795.43

The number of variables (*k*), Akaike’s information criterion for small samples (AIC_c_) the difference in AIC_c_ score relative to the AIC_c_ of the top-ranked model (ΔAIC_c_), the Akaike weight (w_*i*_), and log-likelihood (LogLik) are reported for each model. For brevity, only the top-ranked model, null model, and competing models are shown.

^a^ Abundance is the total number of birds counted per point count.

^b^ The base model which included treatment (mixed seed, Nyjer, or no food [control]), year (Year One [November 2019 –early March 2020] or Year Two [November 2020 –early March 2021]), and the interaction between treatment and year.

^c^ Species Diversity is the Shannon’s Diversity Index value calculated for the bird species detected per point count.

The top-ranked model describing the effects of supplementary feeding on the abundance of granivorous birds in yards contained the main effects of year and treatment, and the interaction between year and treatment (Table 1). The estimates from the top-ranked model suggest that the mean number of granivorous birds was similar in control yards between years (Year One: -0.87, 85% CI = -1.18 –-0.57; Year Two: -0.36, 85% CI = -0.67 –-0.05; Fig 1B). In comparison, there was a significant decrease in the number of granivorous birds between years in mixed seed yards (Year One: 1.38, 85% CI = 1.11–1.64; Year Two: 0.32, 85% CI = 0.02–0.62; Fig 1B). Similarly, the mean number of granivorous birds decreased over the same period in yards provided with Nyjer, but the change was not significant (Year One: 1.26, 85% CI = 1.00–1.52; Year Two: 0.95, 85% CI = 0.66–1.23; Fig 1B). In Year Two when no supplementary food was provided, while the mean number of granivorous birds was equivalent between control and mixed seed yards (control: -0.36, 85% CI = -0.67 –-0.05; mixed seed:0.32, 85% CI = 0.02–0.62; Fig 1B), the mean number of granivorous birds was significantly higher in Nyjer fed yards compared to in both control and mixed seed yards (Nyjer: 0.95, 85% CI = 0.66–1.24).

The top-ranked model describing the effects of supplementary feeding on the abundance of omnivorous birds in yards contained the main effects of year and treatment, and the interaction between year and treatment (Table 1). Estimates from the top-ranked model suggest the mean number of omnivorous birds did not change significantly in control yards between years (Year One: -0.50, 85% CI = -0.81 –-0.19; Year Two: -0.34, 85% CI = -0.66 –-0.02; Fig 1C) or in Nyjer fed yards (Year One: 0.22, 85% CI = -0.07–0.50; Year Two: 0.39, 85% CI = 0.08–0.70; Fig 1C). In contrast, the mean number of omnivorous birds decreased significantly in mixed seed fed yards between years (Year One: 0.77, 85% CI = 0.49–1.06; Year Two: -0.23, 85% CI = -0.56–0.10; Fig 1C). In Year Two when no supplementary food was provided, while the mean number of omnivorous birds was equivalent between control and mixed seed yards (control: -0.34, 85% CI = -0.66 –-0.02; mixed seed: 0.23, 85% CI = -0.56–0.10; Fig 1C), the mean number of omnivorous birds was significantly higher in Nyjer fed yards compared to in control, but not mixed seed yards (Nyjer: 0.39, 85% CI = 0.08–0.70).

Three models describing the effects of supplementary feeding on the abundance of insectivorous birds in yards received equivalent support from the data (Table 1). The top-ranked model contained both ambient temperature and percentage of impervious cover and had the highest Akaike weight (w_*i*_ = 0.34). The second model contained ambient temperature and the third model contained percentage of impervious cover. Both of these models had a lower Akaike weights than the top-ranked model (w_*i*_ = 0.14, w_*i*_ = 0.13, respectively). Given the higher Akaike weight, we considered the top-ranked model as receiving the most support from the data. Estimates from the top-ranked model suggest that ambient temperature and percentage of impervious cover were both significantly associated with the abundance of insectivorous birds (temp: -0.02, 85% CI = -0.02 –-0.01; impervious cover: -2.61, 85% CI = -3.92 –-1.30). The mean number of insectivorous birds did not change significantly in control yards between years (Year One: 0.30, 85% CI = -0.33–0.93; Year Two: 0.48, 85% CI = -0.17–1.13; Fig 1D). Likewise, estimates suggest that the mean number of insectivorous birds did not change significantly between years in mixed seed yards (Year One: 1.39, 85% CI = 0.79–2.00; Year Two: 0.92, 85% CI = 0.29–1.54; Fig 1D) or in yards provided with Nyjer (Year One: 0.78, 85% CI = 0.22–1.34; Year Two: 1.40, 85% CI = 0.83–1.97; Fig 1D). In Year Two when no supplementary food was provided, the mean number of insectivorous birds was similar in yards amongst treatments (mixed seed: 0.92, 85% CI = 0.29–1.54; Nyjer: 1.40, 85% CI = 0.83–1.97; control: 0.48, 85% CI = -0.17–1.13).

### Species diversity

Two models describing the effects of supplementary feeding on species diversity in yards received equivalent support from the data (Table 1). The top-ranked model which contained year, treatment, the interaction between year and treatment, and the percentage of impervious cover had the highest Akaike weight (w_*i*_ = 0.45). The next best supported model had one less parameter and contained year, treatment, and the interaction between year and treatment. However, assessment of model estimates from the top-ranked model suggests that percentage of impervious cover was an important predictor of species diversity. Thus, we considered the top-ranked model as the most supported moving forward.

**Fig 2:**
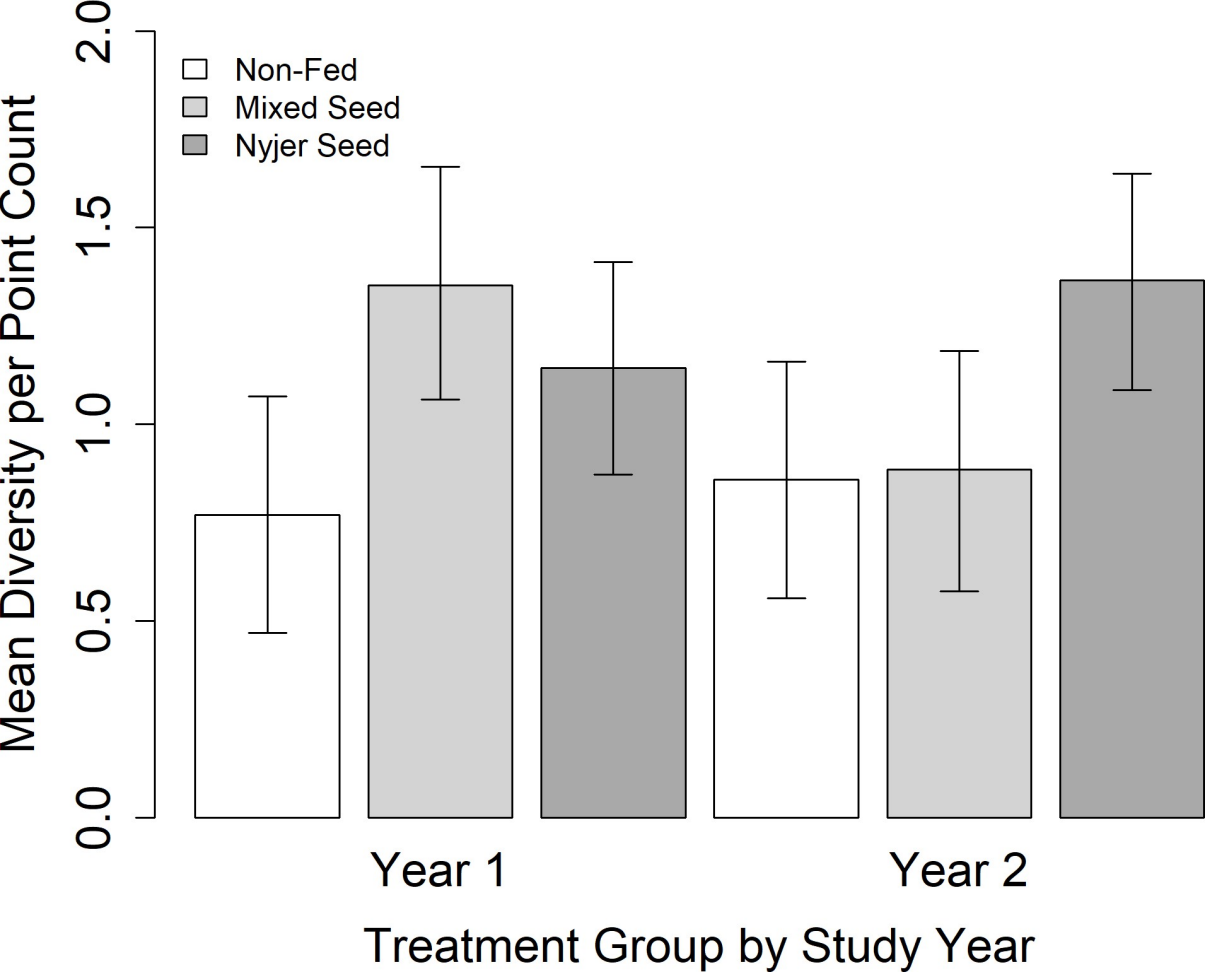
Species diversity of birds (mean ± 85% CI) in response to supplementary feeding in winter. Species diversity was calculated using Shannon’s Diversity Index per point count. In Year One (November 2019 –early March 2020), yards were either provided with either mixed seed, Nyjer, or no food (control). In Year Two (November 2020 –early March 2021), all yards received no food.

Estimates from the top-ranked model suggest that percentage of impervious cover was a significant predictor of species diversity; as the percentage of impervious cover increased, species diversity decreased (-0.77, 85% CI = -1.39 –-0.15). Estimates from the top-ranked model also suggest that species diversity remained similar in control yards between years (Year One: 0.76, 85% CI = 0.47–1.05; Year Two: 0.85, 85% CI = 0.56–1.15; Fig 2). Similarly, species diversity did not differ statistically significantly between years in yards provisioned with Nyjer (Year One: 1.13, 85% CI = 0.87–1.39; Year Two: 1.36, 85% CI = 1.09–1.62; Fig 2). While not statistically significant, there was a tendency for species diversity to be lower in mixed seed yards when food was not provided (Year One: 1.34, 85% CI = 1.06–1.62; Year Two: 0.87, 85% CI = 0.58–1.16; Fig 2). In Year Two when no supplementary food was provided, species diversity was similar in yards amongst treatments (mixed seed: 0.87, 85% CI = 0.58–1.16; Nyjer: 1.36, 85% CI = 1.09–1.62; control: 0.85, 85% CI = 0.56–1.15).

## Discussion

This study is the first to experimentally compare the effects of multiple commonly provided bird foods on the abundance and species diversity of birds in urban yards during winter in a subtropical region. In support of our hypothesis, supplementary feeding increased overall bird abundance, although results were context dependent; Nyjer had no discernable effect on the overall abundance of birds in yards whereas overall bird abundance increased statistically significantly in yards in response to mixed seed. Our results suggest that increases in both granivorous and omnivorous species underscored these results. In contrast, and contrary to our predictions, although species diversity tended to be higher in yards when mixed seed was provided compared to when it was absent, supplementary feeding had no statistically significant effect on species diversity. Rather, species diversity was negatively affected by the percentage of impervious cover within a 500-m radius surrounding focal yards highlighting the importance of maintaining green spaces in cities [54].

### Bird abundance

Our results demonstrating an increase in overall bird abundance in yards in response to supplementary food are consistent with those from prior studies [18,55] showcasing that supplementary feeding is an important predictor of bird abundance, likely because it provides a relatively reliable and aggregated food source. This idea aligns with optimal foraging theory which posits that animals should select foraging sites that optimize net gains in energy and nutrition [18,19]. However, such effects may only be realized when specific food types are provided. In a study of food preferences conducted by Horn et al. [25], visitation rates were higher at feeders containing either black-oil sunflower seed or sunflower chips compared to feeders with other food types (e.g., red milo) for a broad range of species. In comparison, and in similarity to our results, feeders with Nyjer were largely restricted to smaller finches (*Carduelis* spp.). Collectively, these results support our hypothesis that seed type and size may limit use of feeders to specific species based on bill morphology. In comparison, our results demonstrating an increase in overall abundance of birds in the presence of mixed seed suggest that mixed seed blends with a variety of seed types and sizes provide food for a wide range of species that may vary in e.g., nutritional needs or by bill morphology [56].

The effects of mixed seed on the overall abundance of birds in yards was largely driven by an increase in granivorous and omnivorous species. Similarly, Galbraith et al. [22] demonstrated that granivores and omnivores increased when mixed seed and bread were provided in urban yards in New Zealand. Collectively, these results suggest responses to supplementary feeding are likely driven by the foraging ecology of birds where species that are able to exploit the type of food provided (e.g., seed) increase. The absence of an effect of supplementary feeding on the abundance of insectivores, a group of birds represented in our study by species that are not regularly observed at seed feeders during the winter (e.g., yellow-rumped warbler [*Setophaga coronate*], ruby-crowned kinglet [*Regulus calendula*]; [57,58]) corroborate this idea.

Species that experienced the greatest increase in abundance in response to mixed seed included white-winged dove, northern cardinal, house finch (*Haemorhous mexicanus*), and house sparrow (see S1 Table). Apart from the house sparrow, the aforementioned species are native to the US. In comparison, Galbraith et al. [22] noted substantial increases primarily in non-native species in response to supplementary feeding in New Zealand. In addition, and in contrast to our results, they also demonstrated a decrease in insectivorous species likely driven by competition from dominant species attracted to gardens by supplementary food. Collectively, these results suggest responses to supplementary feeding may be site specific, driven by the composition of the avian community and concomitant interactions amongst species.

Surprisingly, the overall abundance of birds remained similar across years in yards in the Nyjer seed treatment group despite the presence of supplementary food in Year One and the absence of supplementary food in Year Two. In addition, the overall abundance of birds was statistically significantly higher in yards in the Nyjer seed treatment group compared to in yards in the control and mixed seed treatment groups in Year Two despite supplementary food being absent from all yards. While we attempted to control for site-specific factors known to affect bird abundance (e.g., feeder density [20], percentage of impervious cover [20,44], presence of water features [42]), it is possible that other determinants of bird abundance such as local habitat composition and structure [33,59] that we were unable to control for could explain our results. Alternatively, potential carry over effects of supplementary feeding during the winter that enhance measures of fitness (e.g., survival, breeding success [26,60]) may explain the higher abundance of birds in Year Two in yards in the Nyjer seed treatment group compared to in yards in the control group. Although not statistically significant, the overall abundance of birds tended to be higher in yards in the mixed seed treatment group compared to those in the control group in Year Two when supplementary food was absent providing further support for this idea.

### Species diversity

Species diversity tended to be higher when supplementary food was provided compared to when it was not provided in yards in the mixed seed treatment group, although the difference was not statistically significant. In comparison, species diversity was equivalent across years of the study in yards in the Nyjer seed treatment group and those in the control group. Previous research has shown that wild birds have preferences for certain food types, influencing their presence at bird feeders [56,61,62]. Such preferences may be underscored by the nutritional content of the seed [56] or by bill shape that influence the types of seed available to a specific species. For example, Horn et al. [25] demonstrated that mourning doves (*Zenaida macroura*) visited white proso millet more than other seed types, likely because the seed’s smaller size made it easier to handle. The mixed seed used in our study contained a variety of seed types and sizes, and thus was likely attractive to a wide range of species resulting in an increase in species diversity. In comparison, the lack of variation in seed type and size in the Nyjer seed provided likely explains the absence of an effect on species diversity.

### Landscape variables

Our results also highlight other important predictors of avian community composition. Specifically, in support of the results from previous studies [54,63,64], species diversity declined as the percentage of impervious cover within a 500-m radius of focal yards increased, likely due to a reduction in suitable habitat for a range of birds. However, the presence of water features in focal yards and feeder density within 100 m of focal yards did not appear to have a significant effect on either species diversity or on the abundance of birds despite previous research demonstrating such effects [20,42]. A possible explanation is our approach; although we considered the density of bird feeders within a 100-m radius of focal yards in our statistical analysis, it is likely that we did not account for all bird feeders present, especially under scenarios where the survey response rate was less than 100%. Similarly, we were unable to account for water features outside of our focal yards which could have influenced the presence of birds. These challenges may, in part explain our results.

## Conclusion

We have showcased that supplementary feeding has pronounced effects on bird communities in urban yards during winter in a subtropical region of the US by significantly increasing the abundance of granivorous and omnivorous birds and by tending to increase species diversity. We have also demonstrated that the effects are specific to the type of food provided. Given our use of relevant and commonly used bird foods, our results can be used to inform feeding recommendations provided by bird food companies and bird food stores to community members, many of whom provide supplementary food to increase backyard birds and to engage in nature [23,24]. However, under scenarios where our results are used in this manner, we stress the importance of acknowledging the potential negative effects of supplementary feeding on wild birds, such as the spread of disease [14,65] which may be especially relevant where the density of bird feeders is high [16]. Further, while some studies report enhanced breeding productivity in response to supplementary feeding [66] others report negative effects [9,11].

Our results provide important insight into the mechanisms underlying variation in bird communities within urban yards and provide support for the idea that supplementary feeding may be a useful tool in promoting ecological urban resilience in response to biodiversity loss following urbanization [67]. However, our results also showcase that the effects of supplementary feeding may be taxa-specific and context dependent. Thus, where supplementary feeding is used as a management tool it should be considered in tandem with other urban management strategies that support diverse bird communities such as the use of green and blue spaces, artificial nesting substrates, and habitat corridors [67]. Because feeding practices vary geographically [3], as do regional species pools, we encourage further study to address the effects of supplementary feeding on the structure of avian communities, especially in understudied regions (e.g., the global south) to further knowledge of the effects of this widespread activity at the global scale.

## Supporting information

S1 Table All identified bird species, with primary dietary guild, detected during point counts used to evaluate the effects of supplementary feeding via three treatment groups (mixed seed, Nyjer seed, no supplementary food [control]) on the abundance and diversity of birds in urban yards in San Antonio, Texas, US between winter 2019–2020 and 2020–2021. (DOCX)
