# Supplementary material for: Supplementary feeding of birds during the winter influences measures of avian community structure in yards in a subtropical city
Source: PLoS One. 2024 May 22;19(5):e0302007. doi: 10.1371/journal.pone.0302007 (PMC11111068; doi:10.1371/journal.pone.0302007)
Supplement: S1 Table — (DOCX) [file pone.0302007.s001.docx]

**S1 Table. All identified bird species, with primary dietary guild, detected during point counts used to evaluate the effects of supplementary feeding via three treatment groups (mixed seed, Nyjer seed, no supplementary food [control]) on the abundance and diversity of birds in urban yards in San Antonio, Texas, US between winter 2019-2020 and 2020-2021.**

| **Species** | **Total Counted** | **Percent Total** | **Dietary Guild** |
| --- | --- | --- | --- |
| Lesser Goldfinch (*Spinus psaltria*) | 805 | 13.2% | Granivore |
| House Sparrow (*Passer domesticus*) | 797 | 13.1% | Omnivore |
| White-winged Dove (*Zenaida asiatica*) | 675 | 11.1% | Granivore |
| Northern Cardinal (*Cardinalis cardinalis*) | 671 | 11.0% | Granivore |
| Black-crested Titmouse (*Baeolophus atricristatus*) | 417 | 6.8% | Insectivore |
| House Finch (*Haemorhous mexicanus*) | 380 | 6.2% | Granivore |
| Carolina Chickadee (*Poecile carolinensis*) | 377 | 6.2% | Omnivore |
| Cedar Waxwing (*Bombycilla cedrorum*) | 269 | 4.4% | Frugivore |
| Carolina Wren (*Thryothorus ludovicianus*) | 220 | 3.6% | Insectivore |
| Northern Mockingbird (*Mimus polyglottos*) | 204 | 3.4% | Omnivore |
| American Goldfinch (*Spinus tristis*) | 163 | 2.7% | Granivore |
| Blue Jay (*Cyanocitta cristata*) | 124 | 2.0% | Omnivore |
| Chipping Sparrow (*Spizella passerina*) | 112 | 1.8% | Granivore |
| Golden-fronted Woodpecker (*Melanerpes aurifrons*) | 88 | 1.4% | Omnivore |
| Unknown^a^ | 82 | 1.3% | – |
| Yellow-rumped Warbler (*Setophaga coronata*) | 81 | 1.3% | Insectivore |
| Orange-crowned Warbler (*Leiothlypis celata*) | 77 | 1.3% | Insectivore |
| Ruby-crowned Kinglet (*Regulus calendula*) | 75 | 1.2% | Insectivore |
| Bewick’s Wren (*Thryomanes bewickii*) | 68 | 1.1% | Insectivore |
| Great-tailed Grackle (*Quiscalus mexicanus*) | 65 | 1.1% | Omnivore |
| European Starling (*Sturnus vulgaris*) | 47 | 0.8% | Omnivore |
| Eastern Phoebe (*Sayornis phoebe*) | 46 | 0.8% | Insectivore |
| Mourning Dove (*Zenaida macroura*) | 46 | 0.8% | Granivore |
| Sparrow^b^ spp. | 28 | 0.5% | – |
| Wren^b^ spp. | 20 | 0.3% | – |
| Downy Woodpecker (*Dryobates pubescens*) | 19 | 0.3% | Omnivore |
| Goldfinch^b^ spp. | 18 | 0.3% | – |
| Ladder-backed Woodpecker (*Dryobates scalaris*) | 18 | 0.3% | Insectivore |
| Inca Dove (*Columbina inca*) | 17 | 0.3% | Granivore |
| Dove^b^ spp. | 11 | 0.2% | – |
| Warbler^b^ spp. | 10 | 0.2% | – |
| Hummingbird^b^ spp. | 9 | 0.1% | Nectivore |
| Pine Siskin (*Spinus pinus*) | 8 | 0.1% | Granivore |
| Hermit Thrush (*Catharus guttatus*) | 6 | 0.1% | Omnivore |
| American Robin (*Turdus migratorius*) | 5 | 0.1% | Omnivore |
| Eurasian Collared Dove (*Streptopelia decaocto*) | 5 | 0.1% | Granivore |
| Red-winged Blackbird (*Agelaius phoeniceus*) | 4 | 0.1% | Granivore |
| Blue-headed Vireo (*Vireo solitarius*) | 3 | 0.0% | Insectivore |
| Golden-crowned Kinglet (*Regulus satrapa*) | 3 | 0.0% | Insectivore |
| Purple Finch (*Haemorhous purpureus*) | 3 | 0.0% | Granivore |
| Curve-billed Trasher (*Toxostoma curvirostre*) | 2 | 0.0% | Omnivore |
| Red-bellied Woodpecker (*Melanerpes carolinus*) | 2 | 0.0% | Omnivore |
| White-throated Sparrow (*Zonotrichia albicollis*) | 2 | 0.0% | Granivore |
| Woodpecker^b^ spp. | 2 | 0.0% | – |
| Brown Thrasher (*Toxostoma rufum*) | 1 | 0.0% | Omnivore |
| Brown-headed Cowbird (*Molothrus ater*) | 1 | 0.0% | Omnivore |
| Dark-eyed Junco (*Junco hyemalis*) | 1 | 0.0% | Granivore |
| White-crowned Sparrow (*Zonotrichia leucophrys*) | 1 | 0.0% | Granivore |
| TOTAL | 6,088 | 100% |  |

The total number of birds observed amongst surveys for each species are provided. Percent total is the percentage for each species of the total number of birds observed. Birds were assigned to dietary guilds granivore, omnivore, insectivore, frugivore, and nectivore based on their winter diet and foraging behavior (birdsoftheworld.org).

^a^ Birds that were detected but could not be identified due to poor visibility or because they were obscured from view.

^b^ Birds that were identified to the family level, but genus and species was uncertain.
